# Diagnosis and therapy of giant epidermoid double cysts with infection on the buttock: A case report and literature review

**DOI:** 10.1097/MD.0000000000037193

**Published:** 2024-02-09

**Authors:** Peiliang Wu, Cong Wang, Yiran Jiang, Zhi Zhang, Junlan Gao, Zhe Fan

**Affiliations:** a Department of General Surgery, the Third People’s Hospital of Dalian, Dalian Medical University, Dalian, China; b Department of Clinical Medicine (2020341116), China Medical University, Shenyang, China; c Department of Cardiology, the Third People’s Hospital of Dalian, Dalian Medical University, Dalian, China; d Department of Critical Care Medicine, The Second Affiliated Hospital of Dalian Medical University Dalian, Liaoning, China; e Liaoning Province Key Laboratory of Corneal and Ocular Surface Diseases Research, The Third People’s Hospital of Dalian, Dalian, China.

**Keywords:** buttock mass, epidermoid cyst, giant, infection, perianal mass, therapy

## Abstract

**Rationale:**

Epidermoid cyst (EC) is a common clinical condition and it can be filled with keratinized material. EC often represents painless, slow progressive growth, and single cyst. The cyst is usually 1 to 5 cm in size. Giant epidermoid cysts on the buttock area are extremely rare, and reports of giant epidermoid double cysts on the buttock are even rarer.

**Patient concerns:**

This paper reports a patient with a painless mass was on the left buttock.

**Diagnosis:**

A giant epidermoid double cysts with infection in a left buttock paranal location.

**Interventions:**

The mass was surgically removed.

**Outcomes:**

The patient recovered well after surgical treatment and currently has no recurrence.

**Conclusion:**

For patients with EC, MRI is recommended as a routine examination before surgery in order to detect the variation and extent of the cyst early. This lays a foundation for the complete resection of the lesion during the operation. The review of relevant literature will hopefully be helpful to clinicians.

## 1. Introduction

Epidermoid cysts (EC) are formed by an apparent or non-apparent external injury that causes the basal cell layer of the epidermis to enter the subcutis and grow further. The cysts are commonly found in areas prone to abrasion or injury, such as the buttocks, elbows, and occasionally at injection sites. Epidermoid cysts located in the buttocks with giant epidermoid cysts are rarely reported in worldwide. Epidermoid cysts with rupture, infection, and formation of double cysts are even rarer. The diagnosis and therapy of a patient with giant epidermoid double cysts located on the left buttock, next to the anus were admitted to our hospital in November 2020. As well as the related literature review are detailed below.

### 1.1. Patient concerns

The patient was a 55-year-old male. 10 years ago, a painless mass was found on the left buttock with no obvious cause. The mass gradually increased in size during the period.

**Figure 1.**
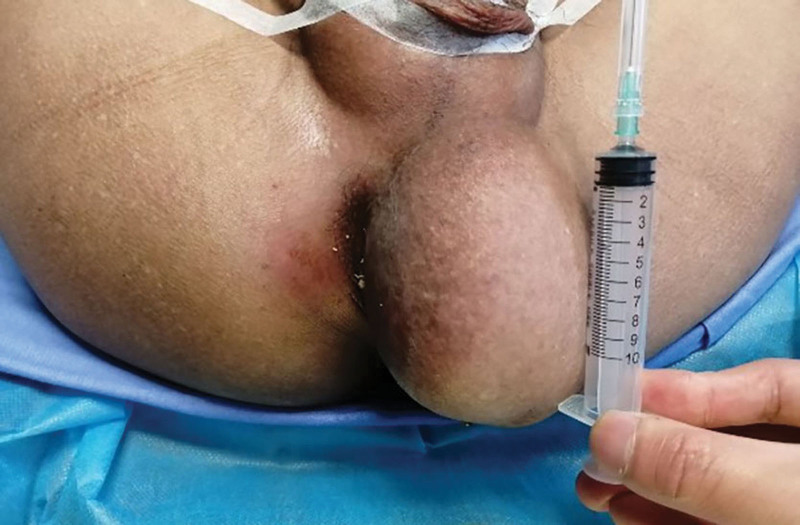
A giant, smooth mass measuring 10 × 7 × 6 cm in size with the anus was squeezed.

Physical examination: A subcutaneous mass was found on the left buttock next to the anus. The size of the mass was about 10 cm × 7 cm × 6 cm (Fig. 1), with soft texture, good mobility, clear boundaries, no tenderness, no skin redness, swelling, or rupture.

**Figure 2.**
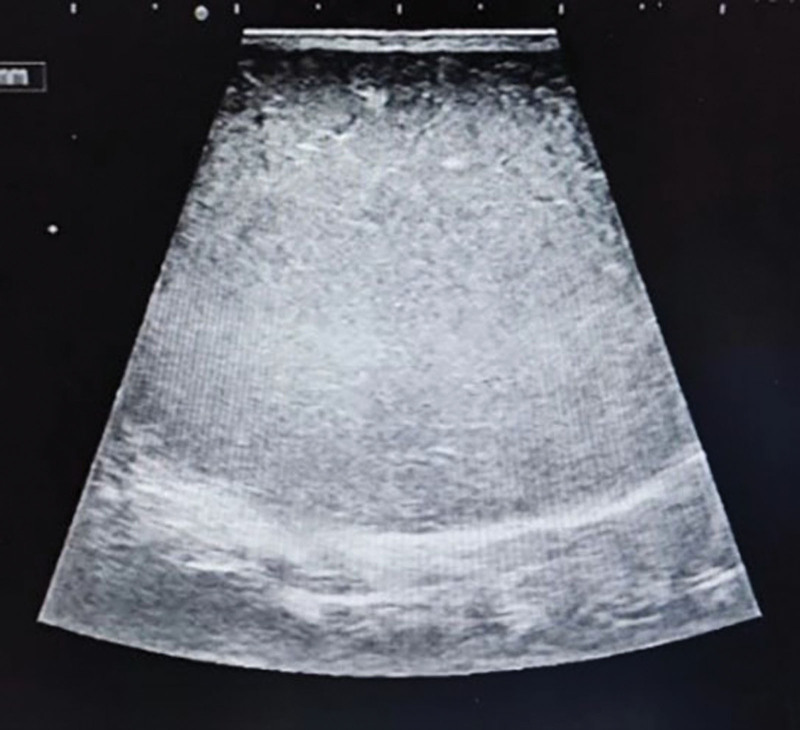
Ultrasound showed a cystic appearance, filled with punctate and flocculen.

**Figure 3.**
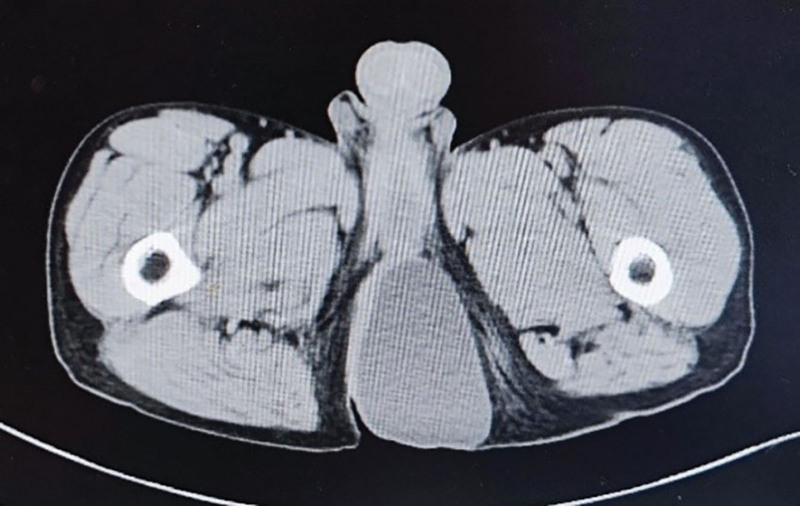
Axial CT image obtained through the pelvis demonstrates a circular cystic predominantly cystic solid shadow, the anus was compressed and shifted to the right. CT = computed tomography.

**Figure 4.**
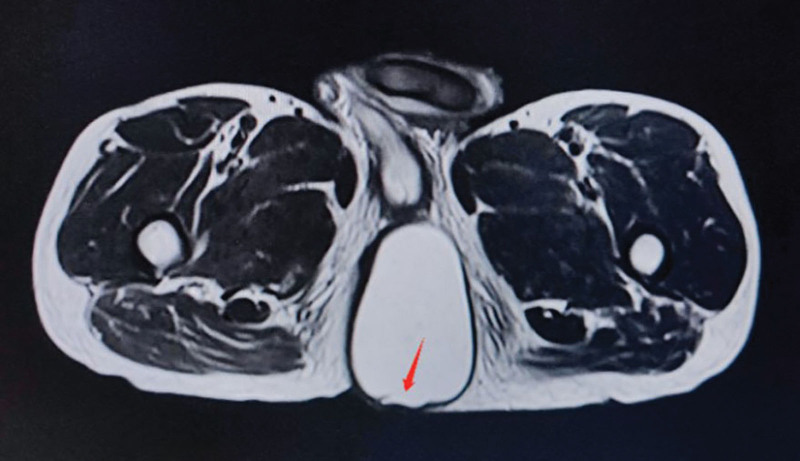
The lesion showed high signal intensity on axial T2W image with irregular backward protrusion of the local posterior wall.

Ancillary examination: ultrasound of the mass (Fig. 2): showed liquid hypoechoic, clear border, uneven echogenicity, filled with punctate and flocculent hypoechogenicity. Color Doppler Flow Imaging (CDFI): no obvious blood signal was seen. The diagnosis suggested that the epidermoid cyst was not excluded. Computed Tomography (CT) scan of the pelvis (Fig. 3): showed a circular cystic predominantly cystic solid shadow in the fatty layer of the left buttock, with clear borders. Right displacement of the anus by pressure. The diagnosis suggested an occupying lesion in the subcutaneous parametrium of the left buttock. The perianal Magnetic Resonance Imaging (MRI) scan (Fig. 4): showed the left buttock subcutaneous paranal fat layer had a round-like long T1 and long T2 signal. The internal signal was inhomogeneous, and a speckled high T1 and low T2 signal shadow was seen. The local posterior wall of the cyst was irregular and slightly protruding backward, showing equal and slightly high mixed signal. The diffusion showed an inhomogeneous high signal shadow. The anus was displaced to the right by pressure. The diagnosis suggested an epidermoid cyst.

### 1.2. Diagnosis

Preoperative diagnosis: left buttock mass (nature to be investigated).

### 1.3. Interventions

**Figure 5.**
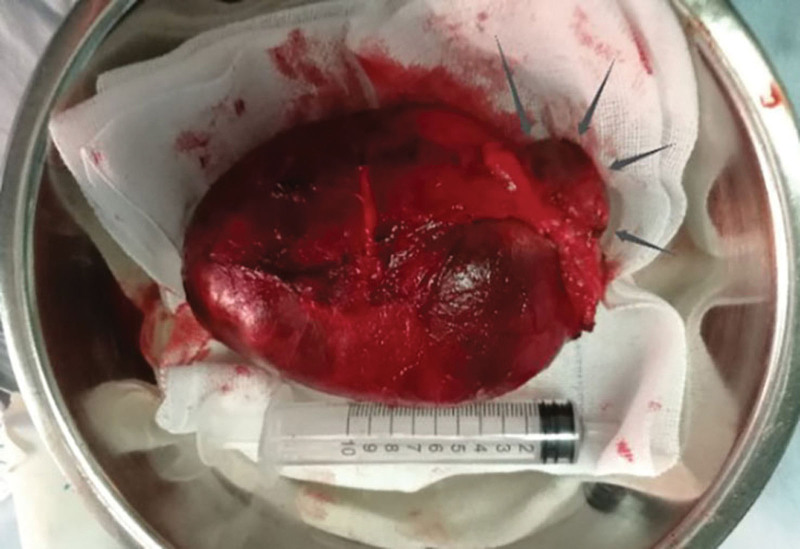
The entire mass was totally excised with a small mass.

Procedure of operation: The patient took the lithotomy position, and the left gluteal mass was excised under continuous epidural anesthesia. The skin and subcutaneous were incised along the long axis of the mass. The mass was seen to be about 10.5 × 7.0 × 6.0 cm in size. Close to the huge mass of a small cyst can be seen, about 2.0 × 2.0 × 1.5 cm in size (Fig. 5). It is considered that the small cyst encapsulated the fluid from rupture and infection of the giant cyst. The medial part of the mass fused with the superficial part of the external anal sphincter, and the lateral part fused with the gluteus maximus fascia. The mass was completely excised and sent for pathology. One negative pressure drainage was placed.

### 1.4. Interventions

**Figure 6.**
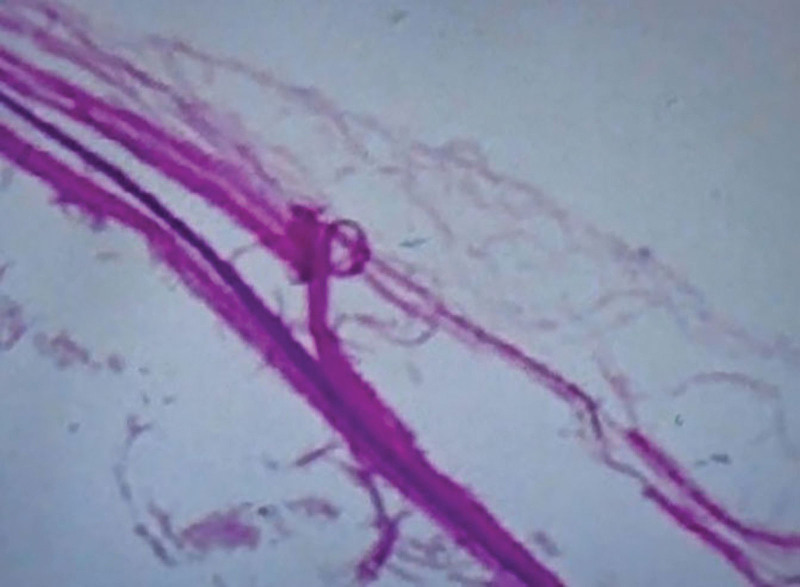
Hematoxilin–Eosin showed an epidermoid cyst with chronic granulomatous inflammation.

Postoperative pathology (Fig. 6): one giant gray nodule was seen at the edge of another small nodule. The giant mass was cystic in a cut section and contained bean residue-like material, while the small nodule was cut and contained grease-like material. The pathological diagnosis suggested: epidermoid cyst with chronic granulomatous inflammation. The patient was discharged on the 22nd postoperative day after giving the incision regular dressing change, decongestion and symptomatic treatment. The incision of the patient recovered well and no abnormality was seen in sitting and walking. There was no recurrence of the epidermoid cyst on the left buttock at the 2-year postoperative follow-up.

## 2. Discussion

An EC is a common benign mass in the skin. In 1835, Cruveilhier first described the epidermoid cyst and named it “tumors perlees.”^[1]^

### 2.1. Epidemiology

Epidermoid cysts are commonly seen in young and middle-aged adults, most of whom are male patients.^[2]^ In recent years, Bashaireh K M and other scholars have found that the incidence of epidermoid cysts was approximately 2:1 in men and women.^[3]^ Although EC often presents as painless, slow-growing, usually 1-5 cm in size.^[4,5]^ Until now, giant epidermoid cysts larger than 5 cm in diameter have rarely been reported clinically.^[6,7]^ EC are mostly solitary, often located in the head and neck,^[8–10]^ trunk,^[11–16]^ buttocks,^[17,18]^ and extremities.^[19]^ EC occasionally occur in the scrotum,^[20]^ thymus,^[21]^ mammary glands,^[22]^ parotid glands^[23]^ and other locations. Multiple EC is rare,^[24]^ often located in the head and face.^[25]^ Most patients have a history of acne.^[26,27]^ Although EC is a common skin lesion, it rarely becomes malignant.^[26,28]^ According to research statistics, the incidence of malignant tumors caused by EC is about 1%.^[2]^ But in recent studies, Kamyab K and other scholars have found that the malignant rate of epidermoid cysts is as high as 9.6%.^[29]^ And the majority of malignant epidermoid cysts are huge. The clinical statistics show that any soft tissue lesion with a maximum diameter greater than 5cm has an approximately 20% chance of malignancy.^[30]^ Through the analysis of the previous literature on the carcinogenesis of epidermoid cysts, there is a great difference in the incidence of carcinogenesis. This may be related to the target population. At the same time, some scholars speculate that epidermoid cysts with chronic inflammation can promote malignant transformation.^[31]^ In this case, the largest diameter of the cyst is about 10.5 cm, and the cyst is accompanied by long-term chronic inflammation. The size and location of the cyst are clinically rare, but the pathological findings did not reveal cancer cells. Although the etiological mechanism of carcinogenesis of epidermoid cyst is unknown, histopathological examination of surgical specimens and long-term postoperative follow-up is necessary for early diagnosis and intervention, so as to significantly improve the prognosis of patients. In this case, the patient recovered well at 2 years postoperative follow-up without recurrence or carcinoma.

### 2.2. Etiology

EC is divided into congenital and acquired.^[26]^ The etiology of congenital epidermoid cysts may be due to abnormal implantation of ectodermal cells during the closure of the neural tube between the third and fifth week of embryonic life.^[32]^ The resulting cysts are most often seen on the embryonic fusion line.^[33]^ The etiology of acquired epidermoid cysts may result from the ectopic transfer of epithelial cells into the deeper tissues of the skin by friction, trauma, surgery, and so on, which gradually proliferate and keratinize.^[34–36]^ Multiple epidermoid cysts are currently studied mostly in relation to genetic history, which can occur in Gardner syndrome caused by a colon gene mutation in adenomatous polyposis,^[37]^ or in Lowe syndrome of X chromosome eye-brain-kidney disease caused by OCLR1 gene mutation. But the exact pattern of mutations has not been elucidated in current studies and needs to be further investigated.^[38,39]^ In recent years, human papillomavirus DNA has been found to be extracted from the scrotum and knees of EC patients.^[40,41]^ Therefore, the human papillomavirus has been considered a potential etiological factor. Some scholars have found that long-term sun injury can also lead to the occurrence of epidermoid cysts in elderly patients.^[42]^ In recent years, Woltsche and Tiwari et al have noted that imiquimod and tacrolimus can also cause epidermoid cysts.^[43,44]^ Although the etiology of epidermoid cysts has been reported to be varied, there is no large-scale systematic study on the specific mechanism. In the present case, the patient was followed up with a history of the prolonged sedentary disease, which may be inextricably linked to the development of epidermoid cysts.

### 2.3. Histopathology

Histologically, the inner layer of the cyst wall of epidermoid cyst is composed of stratified squamous epithelium, the outer layer is supported by collagen tissue. The keratin exfoliates from the epithelium, producing a soft white substance rich in cholesterol crystals to form a cyst. The content of the epidermoid cyst is mostly cheese-like and mostly off-white. Its main components are keratinized substance, a lot of fat, and a small amount of cholesterol.^[5,26,42,45]^ If the inner layer of the cyst is ruptured, the keratin scales in the cyst can overflow into the surrounding soft tissue, resulting in acute foreign body granuloma.^[42]^ If the cyst is infected, there may be a focal accumulation of chronic inflammatory cells outside the cyst wall.^[46]^ In this paper, the pathological diagnosis of the cyst showed that the epidermoid cyst was accompanied by chronic granulomatous inflammation. Combined with the findings during the operation, a small cyst was found close to the giant cyst. We considered the formation of a small cyst around it after rupture and infection. The foreign body in the cyst overflowed and the cyst wall was chronically infected for a long time to form chronic granulomatous inflammation.

### 2.4. Diagnostic imaging

The diagnosis of epidermoid cysts is confirmed primarily by histopathological examination. However, a number of clinical and imaging features, as well as the location of the cyst can be used as a diagnostic basis to help clarify the diagnosis.

Ultrasound: Ultrasound has the advantages of being convenient, inexpensive, and reproducible. It is very helpful in the accurate diagnosis of epidermoid cysts.^[26,47]^ The diagnostic specificity of ultrasound for EC has been reported to be as high as 95.4%.^[48]^ Typical epidermoid cysts have typical cystic features on ultrasound, but they can also become cystic to solid. They have round to oval structures, well-defined borders, and avascular masses in the dermis and subcutaneous tissue. They also have the phenomenon of back acoustic amplification and lateral shadow.^[49,50]^ If the ultrasound shows an “onion-like” appearance, it is largely suggestive of an epidermoid cyst.^[26]^ During the inflammation and rupture phase of the cyst, the blood flow around the cyst is increased, and usually shows low flow.^[46]^ The ultrasound of the patient’s mass in the present case was generally consistent with previous reports in the literature. However, some cysts require further examination due to their location and the atypical ultrasound presentation.

CT: The image appears as a well-wrapped, heterogeneous density mass representing a mixture of fat and keratin, which can calcify internally.^[26]^ The unruptured epidermoid cyst appears as a non-infiltrating, fluid-dense mass with a thin sclerotic wall. The wall can be enhanced on contrast-enhanced images.^[46]^ If the cyst is ruptured, the separation can be seen in the epidermoid cyst. The cyst wall shows thick and irregular enhancement in contrast-enhanced images, and the adjacent soft tissue shows patchy enhancement.^[51]^ In this case, the CT findings of a cystic solid occupying lesion are generally consistent with previous reports in the literature.

MRI: Simple epidermoid cysts showed low signal or equal signal on T1W images and obvious high signal characteristics on T2W images.^[17,50,52]^ The enhanced T1W images showed no enhancement in the center of the mass and thin edge enhancement around the mass. In ruptured cysts, MRI showed a diaphragm, thick and irregular edge enhancement. It’s similar to abscess or bursitis.^[26,47]^ The MRI images of this case were generally consistent with the literature. But the difference was that the local posterior wall was irregular and protruded slightly backward in the image. The image showed an equal and slightly high mixed signal, and the diffuse image showed an irregular high signal. We didn’t notice it. After the operation, we reviewed the image of the MRI which should be a small cyst formed after rupture around the giant cyst. Because the cyst was small and close to the giant cyst, the preoperative ultrasound and CT examination were not described. But only MRI found the small cyst. At the same time, MRI could clearly show the anatomical position of the cyst in relation to the adjacent tissues and organs. The MRI provides an irreplaceable reference for a complete and smooth surgical resection.^[53]^ Therefore, we suggest that MRI examination be performed before the operation for epidermoid cysts to assist in diagnosis and treatment. Especially the cysts with a special shape, complex location, long course of the disease, or infection.

As the ultrasound, CT, and MRI of this patient’s cyst were all suggestive of a cystic mass with a small solid component, it was difficult to perform a preoperative puncture biopsy to obtain tissue from the lesion. The contents of epidermoid cysts were diverse and the locations of the disease were extensive. Therefore, the characteristics of ultrasound were also different, some specific types of cysts showed a lack of specificity in the ultrasound. In addition, they were influenced by the experience of ultrasound physicians, especially the cysts hardened after secondary infection or grew in dark skin. As a result, clinically misdiagnosed epidermoid cysts occurred from time to time.^[54,55]^ In conclusion, sending postoperative specimens for pathology for diagnosis remains an indispensable step.

### 2.5. Differential diagnosis

The common differential diagnoses for epidermoid cysts include Trichilemmal cysts, dermatomal cysts,^[56]^ sebaceous cysts,^[57]^ schwannoma,^[58]^ lipomas,^[59]^ inflammatory masses (furuncle), steatocystoma,^[60,61]^ pilonidal sinus^[61]^ and cutaneous manifestations of Gardner’s syndrome.^[42,62]^ Because the giant cyst in this case is on the buttock, combined with the patient’s previous history of sedentary disease. This disease should also be differentiated from an ischial tuberosity cyst.^[63–65]^

### 2.6. Therapy

Despite the benign and inert nature of epidermoid cysts, complete excision remains our goal for symptomatic lesions. While asymptomatic lesions can remain under conservative observation.^[48]^ When an epidermoid cyst gradually increases in size or has recurrent inflammation that affects daily activities, this is an indication for intervention.^[66]^ Interventions include both non-surgical and surgical treatments. Non-surgical treatment can involve an intralesional injection of triamcinolone acetonide.^[67]^ Surgical treatment techniques include incision and drainage, conventional wide excision (ellipse excision), minimally invasive excision (minimal excision), punch excision, laser excision, and negative pressure suction excision.^[66]^

Conventional wide excision (elliptical excision): The conventional wide excision technique or complete excision of the cyst wall is currently considered the gold standard for the treatment of epidermoid cysts.^[18,36,47,68]^ Because complete excision reduces the probability of recurrence.^[45,69]^ Liu et al^[70]^ found that patients with a history of infection (erythema or swelling) or incisional drainage had thickened cyst walls. Therefore, attention should be paid to the complete resection of the infected part of the cyst walls during the operation so as to reduce the recurrence. Once the cyst has ruptured, it is more difficult to remove it completely.^[18]^ In mildly infected epidermoid cysts (erythema or swelling without systemic symptoms), Meister H et al performed bacterial cultures on extracts from infected cysts and showed that almost half were free of pathogenic bacteria.^[71]^ Therefore, the use of preoperative antibiotics for mildly infected epidermoid cysts needs to be chosen carefully. The anesthesia of this technique mainly uses local anesthetic with epinephrine, which is the preferred method to reduce bleeding. Anesthetics should be injected around the capsule to avoid direct injection into the capsule.^[46]^ However, the gluteal cyst in this case was giant and located beside the anus. In order to reduce the pain and difficulty of the operation, continuous epidural anesthesia was used. The patient reported good during the operation. The diameter of the surgical incision was approximately the same as the cyst.^[18]^ For optimal postoperative recovery, it is important to keep the wound at the line of minimal skin tension. Multiple layers of subcutaneous and superficial sutures will produce the best results.^[26,72]^ However, such time-consuming sutures can sometimes produce significant scarring compared to minimally invasive excision, punch excision, or laser excision.^[26,73]^

Minimally invasive excision (minimal excision), punch excision: The technique of minimally invasive excision and punch excision mainly expose the contents of the cyst through the incision from 2 mm to 5 mm. After the contents of the cyst are drained, the loose capsule is removed through a small incision.^[74]^ The difference is that punch excision requires a disposable dermal perforator to make a small, round incision in the middle of the cyst. Thus it is more convenient for intraoperative manipulation.^[75]^ In past studies, both techniques had the advantages of shorter operative time, less bleeding, faster healing, and reduced scarring. The disadvantage of a slightly higher probability of recurrence than traditional complete excision.^[74]^ As the technique has progressed, in the most recent literature in recent years, Alijanpour et al^[76]^ found that minimally invasive resection in masses ≤ 3 cm in diameter, benign, non-infected cysts had no statistically significant difference in postoperative recurrence rates compared to conventional complete resection, in addition to the previous advantages. In a recent study on the punch excision technique, Cheeley et al similarly found no significant difference in recurrence rates compared to conventional resection for non-infected epidermoid cysts 1 to 3 cm in diameter, except for the previous advantage.^[77]^ However, these 2 techniques have only been studied in small, non-infected epidermoid cysts, the application of large or infected epidermoid cysts is very rare.

Laser excision: Laser excision treatment involves making a small 2-4mm hole in the upper part of the cyst with a CO_2_ laser, applying suitable pressure around the cyst to remove the contents, and then removing the cyst wall using a puncturing method or a hemostatic forceps.^[73]^ Through a retrospective study, Kim et al^[73]^ found that laser excision has the advantages of a shorter operation time, fewer scars, and higher patient satisfaction compared with traditional surgery. At the same time, there is no significant difference in recurrence rate between laser resection and traditional surgery. In a more recent study, Liu et al^[78]^ expanded the range of epidermoid cyst location and mass diameter to 0.2 to 4.0cm, and also found the same advantages of CO_2_ laser excision. However, previous studies have tended to select small epidermoid cysts without infection for laser excision, the treatment of large or infected cysts has not been reported.

Minimally invasive excision combined with laser excision: For minimally invasive treatment combined with laser treatment, the keywords “minimally invasive and laser and epidermal cyst” were searched on PubMed without setting a year limit. Only one study in this field was screened. Fu et al^[79]^ found that minimally invasive combined pulsed dye laser/neodymium-doped yttrium aluminum garnet laser (PDL/Nd: YAG) has the advantages of shorter operative time, smaller scar, and higher patient satisfaction than conventional surgery, the recurrence rate is not statistically significant compared with conventional surgery. However, this study was only a pilot study, the sample size of the study was not large enough, the size and location of the epidermoid cysts were limited, and they were all cysts without infection. The postoperative follow-up time was so short that it was difficult to assess the long-term postoperative recurrence rate. At the same time, whether there was an advantage between this combined treatment technique and simple minimally invasive resection or laser resection needs to be further studied.

Laser excision combined with photodynamic therapy: Photodynamic therapy is considered a promising new approach for the removal of microorganisms including bacteria, fungi, protozoa, and viruses.^[80]^ In a recent research development, Zhang et al^[81]^ innovatively used CO2 laser excision combined with photodynamic therapy for the treatment of infected epidermoid cysts for the first time. It found that the combined treatment was able to inactivate a wide range of microorganisms in the infected focus, including Gram-positive and Gram-negative bacteria. It did not develop drug resistance and damaged adjacent tissues while promoting wound healing and reducing scar formation. Unfortunately, the study did not have a control group, and the number of study subjects was small. There were limitations in the diameter, depth, and location of epidermoid cysts. As well as the most important for the postoperative recurrence rate of infected epidermoid cysts was not counted, which needs to be further explored in depth.

Minimally invasive excision combined with negative pressure suction therapy: This is a minimally invasive approach in which the skin is incised in the middle of the cyst, and the upper cyst wall is bluntly separated. Then the negative pressure suction device is used instead of squeezing them by hand in minimally invasive excision to suck out all the contents of the cyst. Finally, completely separate the cyst wall and remove it.^[82]^ This technique was first proposed by Park et al^[83]^ for the treatment of large or even huge epidermoid cysts. Before that only a few scholars used negative pressure generated by syringes and three-way stopcocks suction for the treatment of smaller cysts. Park et al^[82]^ treated 19 clinical patients with masses with an average diameter of 4.4 cm by using this technique. The results showed that compared with simple minimally invasive excision and punch excision, in addition to the advantages of the latter 2 techniques, such as small scar and short operation time, it also reduced the risk of intraoperative compression cyst rupture and even infection. At the same time, it made up for the deficiency of the current application of minimally invasive excision and punch excision techniques in large or even giant epidermoid cysts. The disadvantage was that larger cysts will produce a dead space due to the removal of cysts, which may lead to the formation of hematoma. However, no follow-up reports of this technique have been seen in the English literature in recent years. Its long-term follow-up recurrence rate and complications need to be investigated. While further clinical studies are needed to determine whether this technique can be applied in patients with deep cyst locations, complex surrounding anatomical relationships, infections, or even ruptures.

To date, most reports on the treatment of epidermoid cysts have focused on minimally invasive treatment in terms of reducing scarring and finding simpler surgical approaches to remove smaller epidermoid cysts.^[78]^ However, little attention has been paid to the treatment of larger epidermoid cysts, especially in cases accompanied by rupture and infection. There are no large-scale studies on the subject. It is now generally accepted that after the resection of a giant epidermal cyst, a skin flap is often required to cover the large defect and eliminate the dead space.^[84]^ Many flaps have been reported in the gluteal region: the V-Y advancement flaps, the gluteus maximus myocutaneous flaps, the adipofascial turnover flaps, and the superior gluteal artery perforator flaps.^[59]^ In this case, the giant cyst was located next to the anus and the anatomy around the cyst was complex. It was difficult to remove it by minimally invasive or punch excision, so the operation was performed by traditional complete resection. It was found that the huge cyst had been broken in the past. Fortunately, after the giant cyst was ruptured, a small cyst had formed around its immediate wall, which was well wrapped. It created the conditions for complete resection of the cyst wall. The incision was closed layer by layer. Postoperative recovery was good and there was no recurrence.

## 3. Conclusion

Epidermoid cysts require epidemiological, etiological, pathophysiological, histopathological, clinical, and imaging assistance in order to make an accurate preoperative diagnosis. Epidermoid cysts need to be treated early because they can gradually increase in size or even become secondary to an infection, leading to changes in sitting position and seriously affect life, it also increases the difficulty of surgery and postoperative scar formation. As far as we know, giant epidermoid cysts are rare, the giant epidermoid cysts located near the anus of the buttocks with ruptured infection have not been reported. The disadvantage is that in this case, since only one case was reported and no control group was set up, the cause and surgical treatment plan were not comprehensively summarized. It is expected that more cases will be reported in the future, and the commonalities and the best treatments will be discovered. In addition, the giant cyst in this case was closely connected with the small cyst. This phenomenon was not described by preoperative ultrasound and CT examination, and was only briefly described by MRI. In this case, in terms of auxiliary examination for double cysts, MRI has nothing in common with ultrasound and CT examination. The surgeon did not confirm the presence of a double cysts until intraoperatively, which undoubtedly increased the uncertainty and risk of the operation. Therefore, it is crucial to perform a thorough radiological ancillary examination for differential diagnosis before surgery. MRI should be performed especially for those with large masses, special locations, irregular cyst shape, or infection. However, there are few clinical studies on huge, ruptured, or infected epidermoid cysts. The study of the imaging manifestations of the double cysts in this case is also very rare. Its latest surgical treatment has only been reported individually. So, we hope that future studies will delve deeper into this direction. In this paper, we reported a case of giant epidermoid double cysts located near the anus of the left buttock with infection and successfully removed completely. For patients with EC, MRI is recommended as a routine examination before surgery in order to detect the variation and extent of the cyst early. We also made a systematic exposition of epidermoid cysts in combination with previous literature. In this case, the variations in the site, clinical presentations, ancillary examination, histopathology, and therapy of the epidermoid cysts have increased the diagnostic and clinical knowledge known to date.

## Author contributions

**Data curation:** Peiliang Wu, Cong Wang.

**Investigation:** Junlan Gao, Zhe Fan.

**Writing – original draft:** Peiliang Wu, Yiran Jiang.

**Writing – review & editing:** Cong Wang, Zhi Zhang, Junlan Gao, Zhe Fan.

## References

[1] Zavanone M Guerra P Rampini PM. A cervico-dorsal intramedullary epidermoid cyst. Case report and review of the literature. J Neurosurg Sci. 1991;35:111–5. 1757803

[2] De Mendonça JCG Jardim ECG Dos Santos CM. Epidermoid cyst: clinical and surgical case report. Ann Maxillofac Surg. 2017;7:151–4. 28713757 10.4103/ams.ams_68_16 PMC5502506

[3] Bashaireh KM Audat ZA Jahmani RA. Epidermal inclusion cyst of the knee. Eur J Orthop Surg Traumatol. 2019;29:1355–8. 30968204 10.1007/s00590-019-02432-4

[4] Takemura N Fujii N Shibata C. Fluid-fluid level in a giant epidermal cyst of the buttock. J Dermatol. 2007;34:193–7. 17291301 10.1111/j.1346-8138.2007.00248.x

[5] Stone MS. Dermatology. Missouri: Mosby Elsevier, 2018:1917–1930.

[6] Sharma R Padhy B. Giant epidermoid cyst: a rarity or negligence? Pan Afr Med J. 2018;30:237. 30574256 10.11604/pamj.2018.30.237.15647 PMC6295305

[7] Lee KI Namgoong S You HJ. Epidemiological characteristics and importance of lobulation of giant epidermal cysts: an 18-year retrospective review of 19 cases. Medicine (Baltimore). 2022;101:e29978. 35945748 10.1097/MD.0000000000029978 PMC9351876

[8] Baskota NP Singh K. Incidental finding of posterior fossa epidermoid, in a head trauma patient: a case report. J Nepalgunj Med Coll. 2019;17:56–7.

[9] Lan C Chen T Huang Y. Scalp epidermoid cyst with abnormal hyperdense on CT scans-a case report and literature review. Interdiscip Neurosurg. 2019;18:100504.

[10] Pupić-Bakrač J Pupić-Bakrač A Bačić I. Epidermoid and dermoid cysts of the head and neck. J Craniofac Surg. 2020;32:347–50 10.1097/SCS.0000000000006834 32796308

[11] Dai DH Hoang PA Thanh CH. Microsurgery for intradural epidermoid cyst at cauda equina level in a 9-year-old child: a case report. Int J Surg Case Rep 2021;82:105932. 33957405 10.1016/j.ijscr.2021.105932 PMC8113878

[12] Maeda T Mishima K Imanishi J. An epidermoid cyst of the thoracic Spine in an elderly patient. World Neurosurg. 2019;127:113–6. 30951916 10.1016/j.wneu.2019.03.262

[13] Maurya VP Singh Y Srivastava AK. Spinal dermoid and epidermoid cyst: an institutional experience and clinical insight into the neural tube closure models. J Neurosci Rural Pract. 2021;12:495–503. 34295103 10.1055/s-0041-1724229 PMC8289537

[14] Sauradeep S Vedantam R. Clinical presentation and surgical outcomes based on age and tumor topography in 59 patients with spinal dermoid cysts. World Neurosurg. 2021;151:e438–e446. 33892167 10.1016/j.wneu.2021.04.048

[15] Sun YC Bong KH Keun KY. Keystone-design perforator island flaps for the management of complicated epidermoid cysts on the back. Sci Rep. 2019;9:14699. 31605009 10.1038/s41598-019-51289-4 PMC6789127

[16] Zafar N Nandan PS Vivek S. Repeated aspiration and sclerotherapy to manage recurrent spinal epidermoid cyst. BMJ Case Rep. 2021;14:e239730. 10.1136/bcr-2020-239730 PMC8276142 34253510

[17] Fukui M Kakudo N Morimoto N. Squamous cell carcinoma arising from an epidermal cyst of the buttock: a case report. Eplasty. 2019;19:ic18. 31666912 PMC6806623

[18] Cui X Wu X Yao X. Surgical treatment for a giant epidermoid cyst on the buttock. Dermatol Ther. 2020;33:e13275. 32061013 10.1111/dth.13275

[19] Beytemür O Yüksel S. Epidermoid cysts localized on extremities. Eklem Hastalik Cerrahisi. 2018;29:27–33. 29526156 10.5606/ehc.2018.58258

[20] Tuncay T Akin SC. Case reports of benign intrascrotal tumors: two epidermoid cysts and one scrotal calcinosis. Arch Ital Urol Androl. 2020;92:64–6. 32255328 10.4081/aiua.2020.1.64

[21] Kim JH Moon JW Kim YN. CT findings of thymic epidermoid cyst in the anterior mediastinum: a case report and literature review. J Korean Soc Radiol. 2022;83:212–7. 10.3348/jksr.2021.0014 PMC9238202 36237357

[22] Zhang Y Song L Zhang H. Giant epidermal inclusion cyst with infection arising within the breast parenchyma: a case report. J Int Med Res. 2021;49:300060521997671. 33730901 10.1177/0300060521997671 PMC8166397

[23] Michal V Samuel H Bronislava D. Rare location of a dermoid cyst in the parotid gland: a case report. Prague Med Rep. 2022;123:193–8. 10.14712/23362936.2022.18 36107448

[24] Nigam JS Bharti JN Nair V. Epidermal cysts: a clinicopathological analysis with emphasis on unusual findings. Int J Trichology. 2017;9:108–12. 28932061 10.4103/ijt.ijt_16_17 PMC5596644

[25] Tchernev G Temelkova I Yungareva I. Multiple epidermal cysts of the scalp: dermatosurgical approach with favourable outcome! Open Access Maced J Med Sci. 2019;7:1509–11. 31198464 10.3889/oamjms.2019.257 PMC6542396

[26] Hoang VT Trinh CT Nguyen CH. Overview of epidermoid cyst. Eur J Radiol Open. 2019;6:291–301. 31516916 10.1016/j.ejro.2019.08.003 PMC6732711

[27] Mayer JE Miller MD Birlea SA. Symmetric multilocular epidermoid cysts on the face: an unusual presentation of a common lesion. JAAD Case Rep. 2018;4:337–9. 29693063 10.1016/j.jdcr.2017.12.014 PMC5911811

[28] Alkul S Nguyen CN Ramani NS. Squamous cell carcinoma arising in an epidermal inclusion cyst. Proc (Bayl Univ Med Cent). 2022;35:688–90. 35991710 10.1080/08998280.2022.2077600 PMC9373787

[29] Kamyab K Kianfar N Dasdar S. Cutaneous cysts: a clinicopathologic analysis of 2,438 cases. Int J Dermatol. 2020;59:457–62. 32034771 10.1111/ijd.14808

[30] De La Hoz Polo M Dick E Bhumbra R. Surgical considerations when reporting MRI studies of soft tissue sarcoma of the limbs. Skeletal Radiol. 2017;46:1667–78. 28884363 10.1007/s00256-017-2745-z

[31] Faltaous AA Leigh EC Ray P. A rare transformation of epidermoid cyst into squamous cell carcinoma: a case report with literature review. Am J Case Rep. 2019;20:1141–3. 31375657 10.12659/AJCR.912828 PMC6690212

[32] Roux A Mercier C Larbrisseau A. Intramedullary epidermoid cysts of the spinal cord. Case report. J Neurosurg. 1992;76:528–33. 1738035 10.3171/jns.1992.76.3.0528

[33] Karmacharya S Sah SK Adhikari S. Epidermoid cyst of the ear lobule in adult. Kathmandu Univ Med J (KUMJ). 2021;19:531–3. 36259203

[34] Suito M Kitazawa T Tsunekawa K. Intertendinous epidermoid cyst of the forearm. Case Reports Plast Surg Hand Surg. 2019;6:25–8. 32002452 10.1080/23320885.2018.1564314 PMC6968711

[35] Babayev R Abbasov B Ekşi M. Thoracic intramedullary epidermoid cyst-timely fashion diagnosis and treatment. Childs Nerv Syst. 2015;31:793–6. 25681950 10.1007/s00381-015-2625-6

[36] Sîrbu OM Chirteş AV Mitricã M. Spinal intramedullary epidermoid cyst: case report and updated literature review. World Neurosurg 2020;139:39–50. 32298825 10.1016/j.wneu.2020.03.207

[37] Koh KJ Park HN Kim KA. Gardner syndrome associated with multiple osteomas, intestinal polyposis, and epidermoid cysts. Imaging Sci Dent. 2016;46:267–72. 28035305 10.5624/isd.2016.46.4.267 PMC5192025

[38] Ikehara S Utani A. Multiple protrusive epidermal cysts on the scalp of a Lowe syndrome patient. J Dermatol. 2017;44:105–7. 27178641 10.1111/1346-8138.13444

[39] Murakami Y Wataya-Kaneda M Iwatani Y. Novel mutation of OCRL1 in Lowe syndrome with multiple epidermal cysts. J Dermatol. 2018;45:372–3. 28516463 10.1111/1346-8138.13881

[40] Kawase M Egawa K Ishiji T. Human papillomavirus type 6/11 identified in an epidermoid cyst of the scrotum. J Dermatol. 2018;45:224–7. 28983946 10.1111/1346-8138.14089

[41] Kawase M Koda M Egawa K. Human papillomavirus type 60 – associated epidermoid cysts recurring in the same location on the knee. J Dermatol. 2022;50:1346–8138. 10.1111/1346-8138.16547 35946322

[42] Zito PM Scharf R. Cyst, Epidermoid (Sebaceous Cyst). 2019.

[43] Woltsche N El-Shabrawi-Caelen L Deinlein T. Eruptive epidermoid cysts after imiquimod treatment of recurrent basal cell carcinoma: a case report. Hautarzt. 2019;70:363–6. 30694354 10.1007/s00105-019-4359-y

[44] Tiwari V Gupta A Gothwal C. Tacrolimus-induced epidermoid cysts in the renal transplant patient. Indian J Nephrol. 2021;31:571–3. 35068767 10.4103/ijn.IJN_318_19 PMC8722548

[45] Cuda JD Rangwala S Taube JM. Fitzpatrick’s Dermatology. New York, NY: McGraw-Hill; 2019:1799–819.

[46] Bin Manie MA Al-Qahtani KH Al Ammar A. Epidermoid cyst of the suprasternal region: a rare case report. Braz J Otorhinolaryngol. 2020;86:133–5. 27320651 10.1016/j.bjorl.2016.04.010 PMC9422676

[47] Brash A Dunham K Wollstein R. Epidermoid cyst in an infected olecranon bursa. J Hand Microsurg. 2020;12:128–9. 32788830 10.1055/s-0040-1701149 PMC7410817

[48] Li J Qian M Huang X. Repeated recurrent epidermoid cyst with atypical hyperplasia: a case report and literature review. Medicine (Baltimore). 2017;96:e8950. 29245264 10.1097/MD.0000000000008950 PMC5728879

[49] Park JW Shin SH Kim BJ. Usefulness of ultrasound in diagnosis of plantar epidermal cyst. Indian J Dermatol Venereol Leprol. 2021;87:872–5. 34491675 10.25259/IJDVL_340_2021

[50] Pressney I Khoo M Hargunani R. Description of the MRI and ultrasound imaging features of giant epidermal cysts. Br J Radiol. 2020;93:20200413. 32755388 10.1259/bjr.20200413 PMC7548353

[51] Nguyen BD Mccullough AE. Ruptured epidermal cyst mimicking cutaneous melanoma on F-18 FDG PET/CT. Radiol Case Rep. 2008;3:125. 27303506 10.2484/rcr.v3i1.125 PMC4896124

[52] Midyett FA Mukherji SK. Epidermoid Cyst. In: Skull Base Imaging. 2020.

[53] Park JW Jeong WG Lee JE. Pictorial review of mediastinal masses with an emphasis on magnetic resonance imaging. Korean J Radiol. 2021;22:139–54. 32783412 10.3348/kjr.2019.0897 PMC7772375

[54] Ma J Zhang YM Zhou CP. Retroperitoneal congenital epidermoid cyst misdiagnosed as a solid pseudopapillary tumor of the pancreas: a case report. World J Clin Cases. 2022;10:2504–9. 35434083 10.12998/wjcc.v10.i8.2504 PMC8968614

[55] Behera B Kumari R Thappa DM. Dermoscopic features of epidermoid cyst beyond punctum. Indian J Dermatol Venereol Leprol. 2022;88:404–8. 35389024 10.25259/IJDVL_670_2021

[56] Balasundaram P Garg A Prabhakar A. Evolution of epidermoid cyst into dermoid cyst: embryological explanation and radiological-pathological correlation. Neuroradiol J. 2019;32:92–7. 30604653 10.1177/1971400918821086 PMC6410456

[57] Solivetti FM Desiderio F Elia F. Sonographic appearance of sebaceous cysts. Our experience and a review of the literature. Int J Dermatol. 2019;58:1353–9. 31209860 10.1111/ijd.14515

[58] Telera S Raus L Vietti V. Schwannomas of the sciatic nerve: a rare and neglected diagnosis. A review of the literature with two illustrative cases. Clin Neurol Neurosurg. 2020;195:105889. 32422470 10.1016/j.clineuro.2020.105889

[59] Niimi Y Takeuchi M Isono N. Squamous cell carcinoma following epidermoid cyst in the buttock. Plast Reconstr Surg Glob Open. 2019;7:e2069. 30881827 10.1097/GOX.0000000000002069 PMC6416116

[60] Chotai N Lim SK. Imaging features of steatocystoma multiplex-back to basics. Breast J. 2021;27:389–90. 33527591 10.1111/tbj.14179

[61] Alotaibi L Alsaif M Alhumidi A. Steatocystoma multiplex suppurativa: a case with unusual giant cysts over the scalp and neck. Case Rep Dermatol. 2019;11:71–6. 31011316 10.1159/000498882 PMC6465725

[62] Charifa A Jamil RT Zhang X. Gardner Syndrome. In: StatPearls. Treasure Island, FL: StatPearls Publishing Copyright © 2022, StatPearls Publishing LLC.; 2022.

[63] Sharma R Tiwari T Goyal S. Typical MRI findings of bilateral ischial bursitis: bilateral Weaver’s bottom. BMJ Case Rep. 2021;14:e246665. 10.1136/bcr-2021-246665 PMC8513221 34642222

[64] Johnson DB Varacallo M. Ischial Bursitis. In: StatPearls. Treasure Island, FL: StatPearls Publishing Copyright © 2022, StatPearls Publishing LLC.; 2022. 29493912

[65] Zhu YC Mi YF Jiang B. Clinical efficacy of arthroscopic treatment in ischial tuberosity cyst: a retrospective comparison study. Medicine (Baltimore). 2022;101:e28128. 35060495 10.1097/MD.0000000000028128 PMC8772662

[66] Sutedja EK Tsaqilah L Sutedja E. An unusual and rare case of generalized multiple epidermoid cysts with a giant epidermoid cyst. Int Med Case Rep J. 2020;13:557–62. 33149705 10.2147/IMCRJ.S276911 PMC7605917

[67] Jakhar D Kaur I. Intralesional drainage injection of triamcinolone acetonide for epidermal cyst. J Am Acad Dermatol. 2018;78:e149–50. 29229576 10.1016/j.jaad.2017.12.008

[68] Zheng J Wang C Liu F. Intraparenchymal epidermoid cyst: proper surgical management may lead to satisfactory outcome. J Neurooncol. 2018;138:591–9. 29532311 10.1007/s11060-018-2826-4

[69] Kumar S Sahana D Rathore L. Fourth ventricular epidermoid cyst - case series, systematic review and analysis. Asian J Neurosurg. 2021;16:470–82. 34660356 10.4103/ajns.AJNS_539_20 PMC8477812

[70] Min HJ Lee JM Han JK. Influence factor in thickness of cyst wall of epidermal cysts. J Craniofac Surg. 2017;28:e369–72. 28328606 10.1097/SCS.0000000000003687

[71] Meister H Taliercio M Shihab N. A retrospective chart review of inflamed epidermal inclusion cysts. J Drugs Dermatol. 2021;20:199–202. 33538555 10.36849/JDD.5014

[72] Shah SHA Wain RAJ Syed S. Step-by-step sebaceous cyst excision: a pictorial guide. Internet J Plast Surg. 2009;7:118–25.

[73] Kim KT Sun H Chung EH. Comparison of complete surgical excision and minimally invasive excision using CO2 laser for removal of epidermal cysts on the face. Arch Craniofac Surg. 2019;20:84–8. 31048644 10.7181/acfs.2018.02152 PMC6505433

[74] Moore RB Fagan EB Hulkower S. Clinical inquiries. What’s the best treatment for sebaceous cysts? J Fam Pract. 2007;56:315–6. 17403333

[75] Lee HE Yang CH Chen CH. Comparison of the surgical outcomes of punch incision and elliptical excision in treating epidermal inclusion cysts: a prospective, randomized study. Dermatol Surg. 2006;32:520–5. 16681659 10.1111/j.1524-4725.2006.32105.x

[76] Alijanpour A Alijanpour K Alijanpour K. Comparison of the surgical outcomes of minimal excision and elliptical excision techniques in treating epidermal inclusion cysts: a prospective randomized study. Shiraz E Med J. 2018:520–5.

[77] Cheeley J Delong Aspey L Mackelfresh J. Comparison of elliptical excision versus punch incision for the treatment of epidermal inclusion cysts: a prospective, randomized study. J Am Acad Dermatol. 2018;79:360–1. 29229572 10.1016/j.jaad.2017.12.011

[78] Liu D Zhou EY Chen D. Epidermoid cyst removal with CO(2) laser fenestration: a retrospective cohort study. J Cosmet Dermatol. 2021;20:1709–13. 33079478 10.1111/jocd.13766

[79] Fu H Luo F Zhao H. A pilot study of minimal invasion combined PDL/Nd:YAG laser in treating facial epidermoid cyst. J Cosmet Dermatol. 2021;20:2805–9. 33569899 10.1111/jocd.13994

[80] Nesi-Reis V Lera-Nonose D Oyama J. Contribution of photodynamic therapy in wound healing: a systematic review. Photodiagnosis Photodyn Ther. 2018;21:294–305. 29289704 10.1016/j.pdpdt.2017.12.015

[81] Zhang LC Hao LM Tan JX. Efficacy of the combination of minimally invasive CO(2) laser incision with photodynamic therapy for infected epidermoid cysts. Photodiagnosis Photodyn Ther. 2020;30:101791. 32344196 10.1016/j.pdpdt.2020.101791

[82] Park SW Choi J Lee HS. Minimal incision suction-assisted excision of a large epidermal cyst. Aesthetic Plast Surg. 2015;39:570–3. 26085226 10.1007/s00266-015-0517-5

[83] Yasuta M Kiyohara T. Negative-pressure suction therapy for epidermal cysts. Dermatol Surg. 2012;38:1751–2. 23030373 10.1111/j.1524-4725.2012.02545.x

[84] Kim SW Yang SH Kim JT. Perforator flaps after excision of large epidermal cysts in the buttocks. Arch Plast Surg. 2014;41:140–7. 24665422 10.5999/aps.2014.41.2.140 PMC3961611

